# Supplementary material for: Field Evaluation of Low-Cost Particulate Matter Sensors in Beijing
Source: Sensors (Basel). 2020 Aug 5;20(16):4381. doi: 10.3390/s20164381 (PMC7472385; doi:10.3390/s20164381)
Supplement: Supplementary file 1 [file sensors-20-04381-s001.pdf]

Supplementary Materials for:

Field Evaluation of Low-Cost Particulate Matter Sensors in Beijing

Han Mei [1,2,3], Pengfei Han [2,*], Yinan Wang [4,*], Ning Zeng [5], Di Liu [2], Qixiang Cai [2], Zhaoze Deng [4], Yinghong Wang [1], Yuepeng Pan [1] and Xiao Tang [1]

1 State Key Laboratory of Atmospheric Boundary Layer Physics and Atmospheric Chemistry, Institute of Atmospheric Physics, Chinese Academy of Sciences, Beijing 100029, China; meihan@mail.iap.ac.cn (H.M.); wangyinghong@mail.iap.ac.cn (Y.W.); panyuepeng@mail.iap.ac.cn (Y.P.); tangxiao@mail.iap.ac.cn (X.T.)
2 State Key Laboratory of Numerical Modeling for Atmospheric Sciences and Geophysical Fluid Dynamics, Institute of Atmospheric Physics, Chinese Academy of Sciences, Beijing 100864, China; liudi@mail.iap.ac.cn (D.L.); caiqixiang@mail.iap.ac.cn (Q.C.)
3 College of Earth and Planetary Sciences, University of Chinese Academy of Sciences, Beijing 100049, China
4 Laboratory of Middle Atmosphere and Global Environment Observation, Institute of Atmospheric Physics, Chinese Academy of Sciences, Beijing 100029, China; dengzz@mail.iap.ac.cn
5 Department of Atmospheric and Oceanic Science, and Earth System Science Interdisciplinary Center, University of Maryland, College Park, MD 20742. USA; zeng@umd.edu
* Correspondence: pfhan@mail.iap.ac.cn (P.H.); wangyinan@mail.iap.ac.cn (Y.W.)

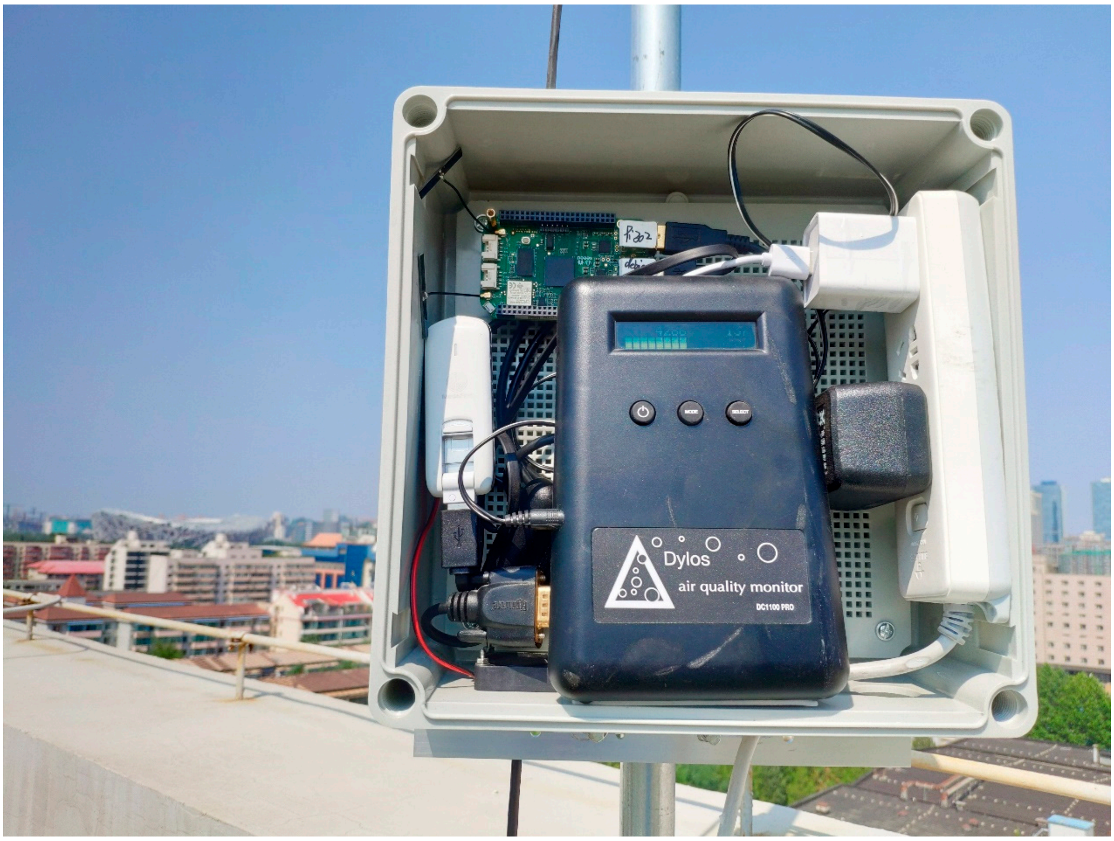

Figure S1. Photo of Dylos DC1700 at the Beijing (IAP, CAS) site.

Table S1. Record of statistical variables shown on the boxplot in Figure 6.

PM2.5 range	0~35	35~75	75~115	115~150	150~250	>250
count	12383	6764	2142	626	1170	15
mean	10.84	-5.64	-10.61	-11.38	-14.88	-17.09
std	89.45	30.30	27.63	25.36	22.02	17.96
min	-100.00	-98.33	-88.81	-88.40	-97.25	-37.96
25%	-40.13	-22.68	-31.42	-26.57	-29.18	-26.00
50%	0.50	-5.79	-12.60	-14.54	-17.61	-24.82
75%	38.00	11.10	6.34	4.62	-1.23	-12.25
max	1999.00	199.54	118.75	82.43	65.34	21.86

std: standard deviation;

25%,50%,75%: 25th ,50th and 75th percentile values (the interquartile range)

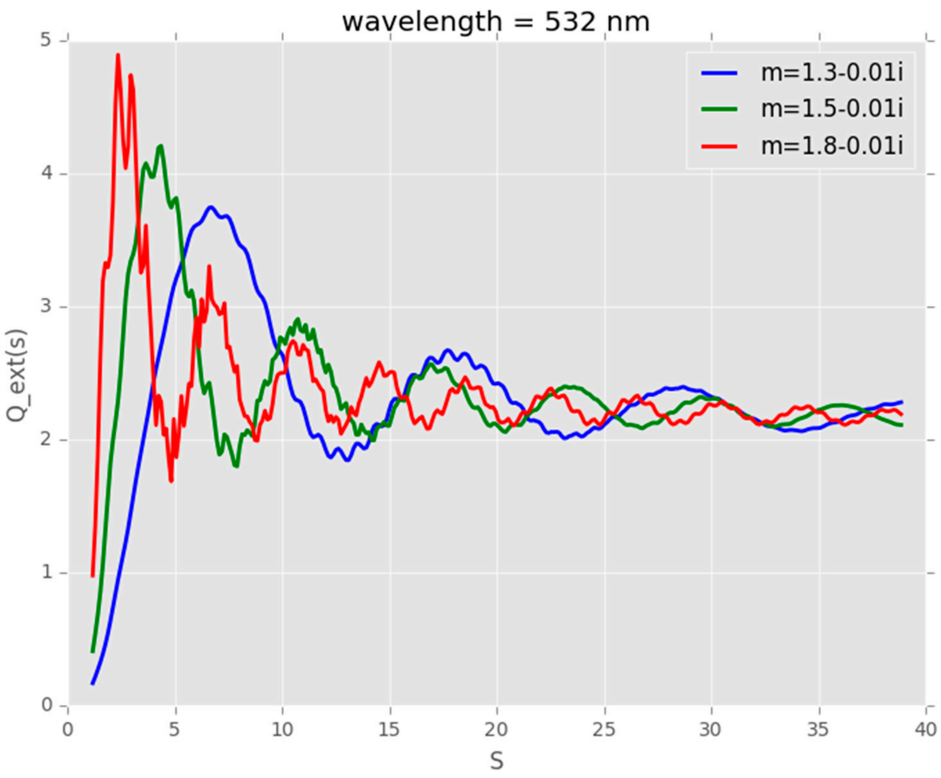

Figure S2. Aerosol extinction efficiency factor calculated by Mie theory. The x axis is scale factor equal to 2πr/λ， m is complex refractive index. The y axis is aerosol extinction efficiency factor.

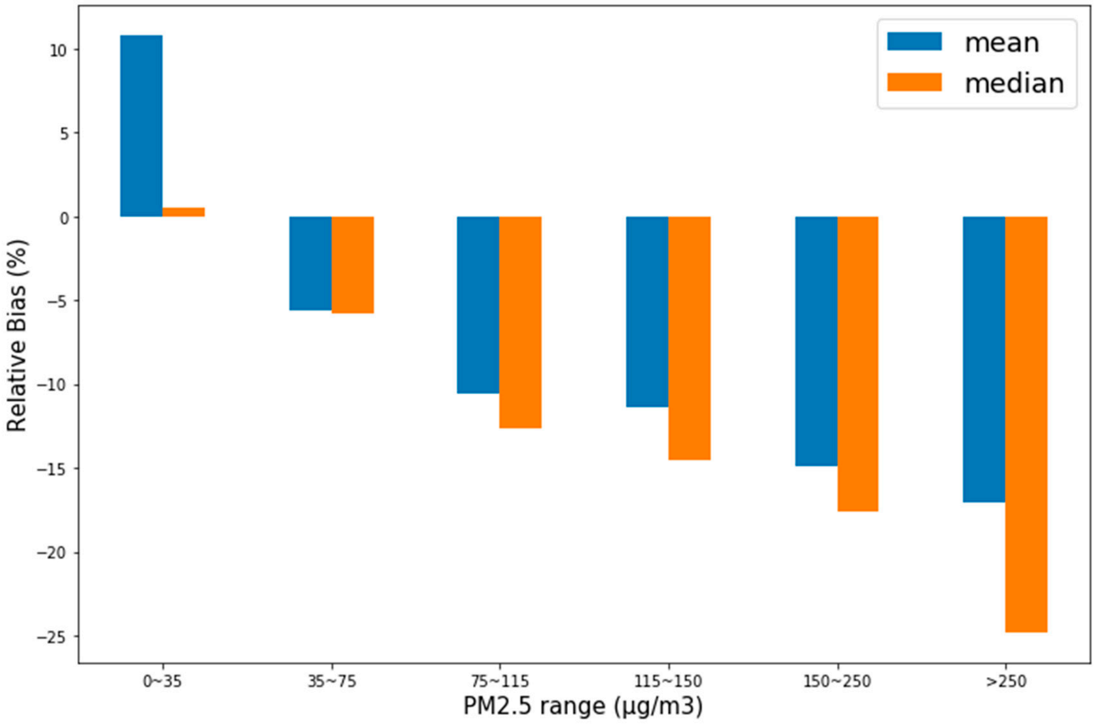

Figure S3. Histogram of average relative bias (mean) and median relative bias (median) of PMSA003 sensors under different concentrations.

Table S2. Record of statistical variables shown on the boxplot in Figure 8.

RH range	0~20	20~40	40~60	60~75	>75
count	4239	10229	3645	1332	258
mean	-3.74	-1.58	0.00	8.55	15.02
std	9.54	11.23	14.08	15.41	17.68
min	-56.30	-68.00	-63.87	-33.85	-24.93
25%	-6.68	-6.49	-7.54	-0.90	2.17
50%	-2.23	-0.42	-0.15	7.96	14.71
75%	1.20	4.27	7.19	16.59	26.42
max	35.23	195.04	299.64	74.51	59.51

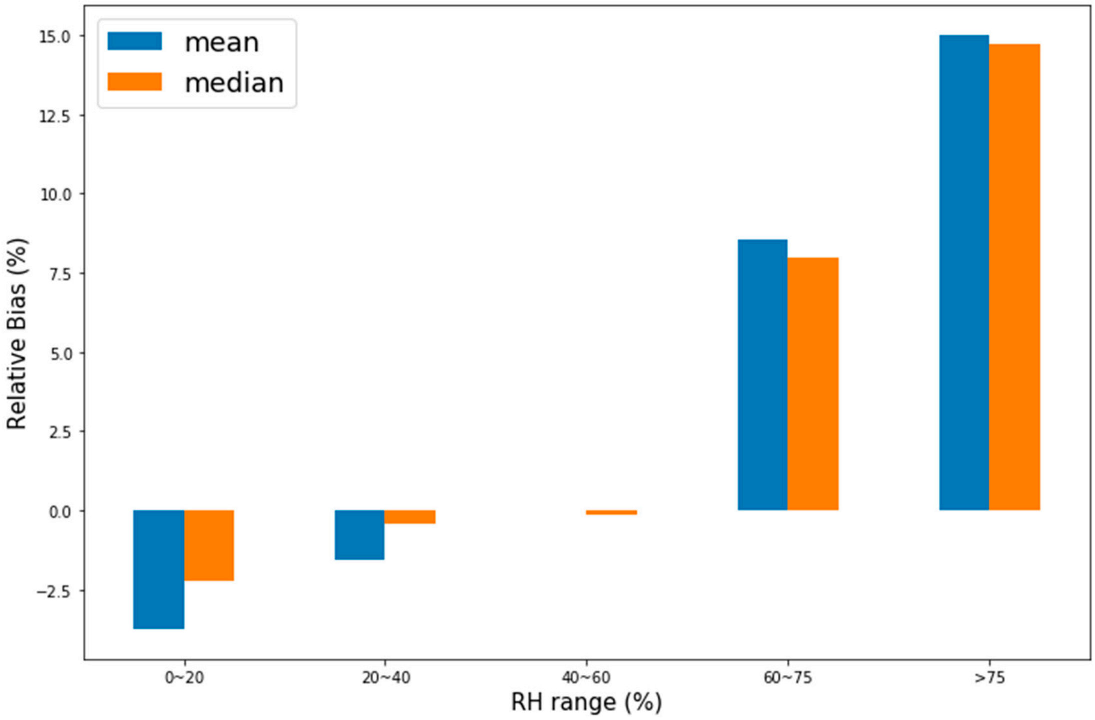

Figure S4. Histogram of average relative bias (mean) and median relative bias (median) of PMSA003 sensors under different relative humidity (RH) ranges.
